# CUDASW++ 3.0: accelerating Smith-Waterman protein database search by coupling CPU and GPU SIMD instructions

**DOI:** 10.1186/1471-2105-14-117

**Published:** 2013-04-04

**Authors:** Yongchao Liu, Adrianto Wirawan, Bertil Schmidt

**Affiliations:** 1 Institut für Informatik, Johannes Gutenberg Universität Mainz, Mainz, Germany

**Keywords:** Smith-Waterman, CUDA, GPU, PTX SIMD instructions, Concurrent execution

## Abstract

**Background:**

The maximal sensitivity for local alignments makes the Smith-Waterman algorithm a popular choice for protein sequence database search based on pairwise alignment. However, the algorithm is compute-intensive due to a quadratic time complexity. Corresponding runtimes are further compounded by the rapid growth of sequence databases.

**Results:**

We present CUDASW++ 3.0, a fast Smith-Waterman protein database search algorithm, which couples CPU and GPU SIMD instructions and carries out concurrent CPU and GPU computations. For the CPU computation, this algorithm employs SSE-based vector execution units as accelerators. For the GPU computation, we have investigated for the first time a GPU SIMD parallelization, which employs CUDA PTX SIMD video instructions to gain more data parallelism beyond the SIMT execution model. Moreover, sequence alignment workloads are automatically distributed over CPUs and GPUs based on their respective compute capabilities. Evaluation on the Swiss-Prot database shows that CUDASW++ 3.0 gains a performance improvement over CUDASW++ 2.0 up to 2.9 and 3.2, with a maximum performance of 119.0 and 185.6 GCUPS, on a single-GPU GeForce GTX 680 and a dual-GPU GeForce GTX 690 graphics card, respectively. In addition, our algorithm has demonstrated significant speedups over other top-performing tools: SWIPE and BLAST+.

**Conclusions:**

CUDASW++ 3.0 is written in CUDA C++ and PTX assembly languages, targeting GPUs based on the Kepler architecture. This algorithm obtains significant speedups over its predecessor: CUDASW++ 2.0, by benefiting from the use of CPU and GPU SIMD instructions as well as the concurrent execution on CPUs and GPUs. The source code and the simulated data are available at http://cudasw.sourceforge.net.

## Background

The Smith-Waterman (SW) algorithm [1,2] is a dynamic-programming-based approach to identify optimal local alignments of biological sequence pairs. Due to its maximal sensitivity for local alignments, this algorithm is a fundamental operation in bioinformatics, including biological sequence database search, multiple sequence alignment [3,4] and next-generation sequencing read alignment [5,6]. In biological sequence database search, the similarities between sequences can be inferred from optimal local alignment scores calculated by the SW algorithm. To calculate optimal local alignment scores, the SW algorithm has a linear space complexity and a quadratic time complexity. However, this quadratic time complexity makes the SW algorithm computationally demanding for large-scale sequence database search. This is further compounded by the rapid growth of sequence databases.

Therefore, several heuristics such as FASTA [7] and BLAST [8,9] have been proposed to accelerate the sequence database search, but not guaranteeing to discover optimal local alignments. These heuristics usually produce considerably good results, but might fail to detect some distantly related sequences due to the loss of sensitivity. Hence, it has great significance to accelerate the SW algorithm so as to maintain optimal results. Consequently, a lot of efforts have been made to parallelize this computation on high-performance computing architectures ranging from loosely-coupled to tightly-coupled ones. Architecture examples include clouds [10], clusters [10] and accelerators [11]. Recent acceleration approaches focus on the use of field programmable gate arrays (FPGAs), single instruction multiple data (SIMD) vector execution units on CPUs, multi-core Cell Broadband Engine (Cell/BE), and many-core general-purpose GPUs, especially based on the compute unified device architecture (CUDA)-enabled GPUs.

For FPGAs, some approaches based on linear systolic arrays and custom instructions have been proposed. Oliver *et al*. [12,13] constructed a linear systolic array on a standard Virtex II FPGA board to perform the SW algorithm with affine gap penalties. Li *et al*. [14] designed custom instructions to support massively parallel computing of the SW algorithm on an Altera Stratix EP1S40 FPGA.

For SIMD vector execution units on CPUs, most efforts have been concentrated on intra-task parallelization that accelerates the alignment of a single sequence pair. Intra-task parallelization approaches can be generally classified into two computational patterns: (*i*) SIMD vectors parallel to minor diagonals in the alignment matrix [15], and (*ii*) SIMD vectors parallel to the query sequence by means of either a sequential [16] or a striped layout [17]. The former pattern is runtime independent of scoring schemes, but has complicated data management and limited speedups. The latter pattern proves to be faster, but is runtime sensitive to scoring schemes. Besides intra-task parallelization, some approaches based on inter-task parallelization have also been investigated [18,19].

Unlike intra-task parallelization, the inter-task parallelization computes multiple alignments in parallel in a SIMD vector. The major advantages of this parallelization are the independence between alignments in SIMD vectors and the runtime independence of scoring schemes. These two parallelization approaches provide a general framework for other architectures with SIMD vector execution units, such as Cell/BEs and general-purpose GPUs. Cell/BE is a heterogeneous multi-core architecture comprised of one general-purpose power processor element and eight synergistic processing elements that serve as SIMD accelerators. On Cell/BEs, several approaches have been implemented [20]–[22], all of which are designed based on the striped approach [17]. For general-purpose GPUs, Liu *et al*. [23] developed an initial OpenGL-based implementation. With the emergence of CUDA programming model, several implementations targeting different generations of CUDA-enabled GPU architectures [24]–[29] have been developed using CUDA, among which CUDASW++ 2.0 [26] is one of the fastest.

In this paper, we present CUDASW++ 3.0, which yields faster SW protein database search by coupling CPU and GPU SIMD instructions and conducting concurrent CPU and GPU computations. Similar approaches of coupling CPU and GPU computation have been investigated in [30] and [31] for phylogeny-aware alignment kernel and short-read alignment, respectively. To balance the runtimes of CPU and GPU computations, we have dynamically distributed all sequence alignment workloads over CPUs and GPUs, as per their compute power. For the computation on CPUs, we have employed the streaming SIMD extensions (SSE)-based vector execution units and multi-threading to speed up the SW algorithm. For the computation on GPUs, for the first time, we have investigated a GPU SIMD parallelization approach using PTX SIMD video instructions. Using the PTX SIMD instructions, we can obtain more data parallelism on GPUs beyond the single instruction multiple thread (SIMT) execution model implemented such as in CUDASW++ 2.0.

We have evaluated the performance of CUDASW++ 3.0 and three other top-performing algorithms: CUDASW++ 2.0, SWIPE [19] and BLAST+ [32] using both the Swiss-Prot protein database and a simulated database comprised of equal-length sequences. Two Kepler-based graphics cards, namely GeForce GTX 680 (GTX680) and GeForce GTX 690 (GTX690), have been used for all evaluations of our algorithm. On a GTX680 (GTX690), CUDASW++ 3.0 achieves a maximal performance improvement of 2.9 (3.2) times over CUDASW++ 2.0 using the Swiss-Prot database and of 2.2 (2.3) times using the simulated database. Furthermore, our algorithm gains an average performance of 109.4 (169.7) billion cell updates per second (GCUPS), with a maximum of 119.0 (185.6) GCUPS, on the Swiss-Prot database and an average performance of 118.0 (196.2) GCUPS, with a maximum of 121.6 (204.7) GCUPS, on the simulated database. In addition, CUDASW++ 3.0 demonstrated significant speedups on average over SWIPE and BLAST+.

### The Smith-Waterman algorithm

Given a sequence *S*, define *S*[*i*] to denote the *i*^th^ residue of *S*, and *S*_*i*_ to denote the prefix of *S* ending at position *i*. Given two sequences *S* and *T*, the recurrence of the SW algorithm with affine gap penalties is defined as

(1) $$\begin{array}{l} H_{i,j}=\max\{H_{i-1,j-1}+M(S\left[i\right],T[j]),E_{i,j},F_{i,j},0\}\\ E_{i,j}=\max\left\{E_{i-1,j}-\beta ,H_{i-1,j}-\alpha \right\}\\ F_{i,j}=\max\left\{F_{i,j-1}-\beta ,H_{i,j-1}-\alpha \right\} \end{array}$$

where *H*_*i*,*j*_, *E*_*i*,*j*_ and *F*_*i*,*j*_ represent the local alignment score of two prefixes *S*_*i*_ and *T*_*j*_ with *S*[*i*] aligned to *T*[*j*], *S*[*i*] aligned to a gap and *T*[*j*] aligned to a gap, respectively. *M* is the scoring matrix which defines the substitution scores between residues, α is the sum of the gap open and extension penalties, and β is the gap extension penalty. The recurrence is initialized as *H*_*i*,0_ = *H*_0,*j*_ = *E*_*0*,*j*_ = *F*_*i*,0_ = 0 for 0≤*i*≤|*S*| and 0≤*j*≤|*T*|. The optimal local alignment score is the maximal alignment score in the alignment matrix *H* and can be calculated in linear space.

### GPU architecture

CUDA-enabled GPUs have evolved into highly parallel many-core processors with tremendous compute power and very high memory bandwidth. They are especially well-suited to address computational problems with high data parallelism and arithmetic density. A CUDA-enabled GPU can be conceptualized as a fully configurable array of scalar processors (SPs). These SPs are further organized into a set of streaming multiprocessors (SMs) under three architecture generations: Tesla [33], Fermi [34] and Kepler [35]. Since our algorithm targets the newest Kepler architecture, it is fundamental to understand the features of the underlying hardware and the associated parallel programming model.

For the Kepler architecture, each SM comprises 192 CUDA SP cores sharing a configurable 64 KB on-chip memory. The on-chip memory can be configured at runtime as 48 KB shared-memory with 16 KB L1 cache, 32 KB shared-memory with 32 KB L1 cache, or 16 KB shared-memory with 48 KB L1 cache, for each CUDA kernel. This architecture has a local memory size of 512 KB per thread and has a L1/L2 cache hierarchy with a size-configurable L1 cache per SM and a dedicated unified L2 cache of size up to 1,536 KB. However, L1 caching in Kepler is reserved only for local memory accesses such as register spills and stack data. Global memory loads can only be cached in L2 cache and the 48 KB read-only data cache [36]. Same as all previous architectures, threads launched onto a GPU are scheduled in groups of 32 parallel threads, called warps, in SIMT fashion.

To facilitate general-purpose data-parallel computing, CUDA-enabled GPUs have introduced PTX, a low-level parallel thread execution virtual machine and instruction set architecture (ISA) [37]. PTX provides a stable programming model and ISA that spans multiple GPU generations. For the Kepler architecture, SIMD video instructions are introduced in PTX, which operate either on pairs of 16-bit values or quads of 8-bit values. These SIMD instructions expose more data parallelism of GPUs and provide an opportunity for us to achieve higher speed for data-parallel compute-intensive problems. In this paper, we have explored PTX SIMD instructions to further accelerate the SW algorithm on Kepler-based GPUs.

## Methods

### Program outline

**Figure 1:**
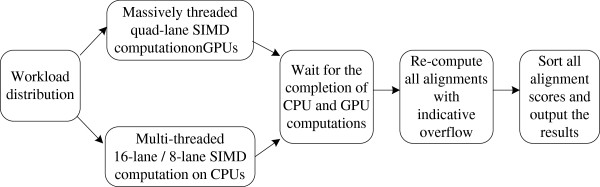
Program workflow of CUDASW++ 3.0.

CUDASW++ 3.0 gains high speed by benefiting from the use of CPU and GPU SIMD instructions as well as the concurrent CPU and GPU computations. Our algorithm generally works in four stages: (*i*) distribution of workloads over CPUs and GPUs according to their compute power; (*ii*) concurrent CPU and GPU computations; (*iii*) re-computation of all alignments that have exceeded the 8-bit accuracy using CPU 8-lane 16-bit SIMD instructions; and (*iv*) sorting of all alignment scores in descending order and output the results. Figure 1 illustrates the workflow of our algorithm. In our algorithm, all subject sequences are pre-sorted in ascending order of sequence length.

### Workload distribution

Our workload distribution in Stage (*i*) balances the runtimes between the CPU and GPU SIMD computation. Hence, the compute power of CPUs and GPUs should be taken into consideration in order to generalize our approach to different hardware configurations. Our distribution policy calculates a rate *R* of the number of residues from the database assigned to GPUs, which is calculated as

(2) $$R=\frac{N_{G}f_{G}}{N_{G}f_{G}+N_{C}f_{C}/C}$$

where *f*_*C*_ and *f*_*G*_ are the core frequencies of CPUs and GPUs, *N*_*C*_ and *N*_*G*_ are the number of CPU cores (i.e. threads) and the number of GPU SMs, and *C* is a constant derived from empirical evaluations, i.e. 3.2 and 5.1 for the query profile and its variant, respectively. When using multiple GPUs, our algorithm assumes that they have the same compute power and will calculate *N*_*G*_ by summing up the number of SMs on all GPUs.

After obtaining *R*, we calculate the number *N*_*R*_ of residues assigned to GPUs as *R* times the total number of residues in the database. Subsequently, all subject sequences assigned to GPUs can be determined by summing the sequence lengths in ascending order until it reaches *N*_*R*_. All other subject sequences will be distributed to CPUs.

### CPU SIMD computation

In Stage (*ii*), the CPU SIMD computation consists of two steps. First, we compute the SW algorithm by splitting an SSE vector to 16 lanes with 8-bit lane width. This allows aligning a query in parallel to 16 subject sequences following the inter-task parallelization model. Secondly, we re-compute all alignments, whose scores have overflow potential, using 8-lane SSE vectors with 16-bit lane width. We determine an alignment to have overflow potential by comparing its score with a score limit calculated by subtracting from 128 the maximum substitution score in the scoring matrix. If the score ≥ the score limit, the alignment is deemed to have an overflow potential and thus requires re-computation. Our approach is based on the open-source SWIPE and more details about the specific implementation of the SSE-based SW algorithm can be obtained from [19].

In our algorithm, users are allowed to use multiple threads to conduct the CPU SIMD computation. Since the workload (i.e. subject sequences assigned to CPUs) is known beforehand, we calculate the total number of residues in all assigned subject sequences and (nearly) equally distribute all residues over all threads using a sequence as a unit. This distribution aims to make each thread hold (roughly) the same number of residues, but not necessarily receiving the same number of subject sequences.

### GPU SIMD computation

#### Core PTX SIMD assemblies

**Figure 2:**
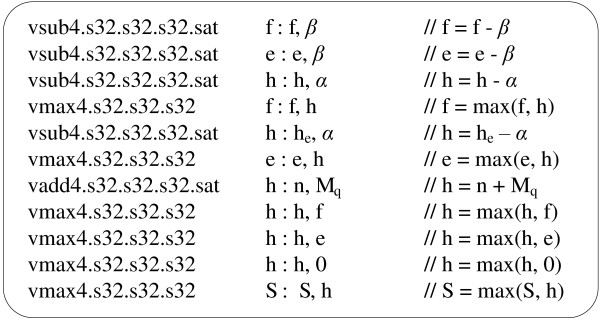
Code in CUDA PTX SIMD assemblies.

We have implemented the recurrence in Equation (1) with PTX SIMD assembly instructions. The code consists of ten assembly instructions for the recurrence and one instruction for obtaining the optimal local alignment score. Figure 2 shows the PTX SIMD assembly instructions.

The figure shows that every instruction operates on quads of 8-bit signed values, corresponding to four independent alignments. Variables *h*, *n* and *h*_*e*_ represent the alignment score vectors corresponding to matrix *H*, where *h* denotes the score vector of the four current cells, *n* the score vector of the four diagonal neighbours and *h*_*e*_ the score vector of the four upper neighbours. Variables *e* and *f* represent the score vectors corresponding to the matrices *E* and *F* respectively, and *S* stores the current maximum alignment scores. For additions and subtractions, saturation instructions have been used to clamp the values to their appropriate signed ranges.

#### CUDA-enabled parallelization

For quad-lane SIMD computing on GPUs, four adjacent subject sequences (in the pre-sorted list as mentioned above) are assigned to a single thread, with each vector lane corresponding to each sequence. To facilitate data fetches for SIMD vectors, a two-dimensional sequence profile of size 4×*l* will be created for four sequences, where *l* is the maximum length of the four sequences. In a sequence profile, each row is a quad-lane residue vector represented as an *integer* data type, and is created by packing four residues of the same index in their corresponding sequences with each residue occupying 8 bits. To reduce the number of texture fetches, we have further packed four successive residue vectors using a *uint4* vector data type for each sequence profile. Thus, we can realize four residue vectors for four subject sequences by a single texture fetch. Using a profile as a unit, we store all profiles in the texture memory following the same layout as in CUDASW++ 2.0.

For the linear-space SW algorithm, we require two intermediate buffers to store one row for matrices *H* and *E* (in our case) respectively. Instead of global memory, we have allocated them in local memory. Since the Kepler architecture has 512 KB per-thread local memory, theoretically we can support subject sequences as long as 65,536 on GPUs. Our algorithm sets the maximum subject sequence length to be 3,072 by default, but allows users to configure it at compile time because the two intermediate buffers have to be statically allocated in local memory.

The sequence length deviation generally causes runtime imbalance between threads, which in return can waste GPU compute power. In this regard, we have developed two CUDA kernels based on two parallelization approaches: static scheduling and dynamic scheduling. These two kernels are invoked at runtime based on the sequence length deviation of the database. For both approaches, we compute the total number of thread blocks from the total number of sequence profiles, which is constructed from the workload assigned to the GPU. Since each thread has its own intermediate buffers, the static scheduling parallelization launches all thread blocks onto the GPU at the same time, which is common for launching a CUDA kernel. The parallelization will rely on the CUDA runtime system to maximize the utilization of computational resources of GPUs. Besides the CUDA runtime system, the dynamic scheduling approach attempts to intervene with the scheduling of thread blocks on GPUs. This parallelization launches a small set of thread blocks of size *N*_*T*_ to carry out the whole computation, regardless of the assigned workload. *N*_*T*_ is defined as:

(3) $$N_{T}=\frac{2N_{\mathrm{SM}}\times N_{\mathrm{MRT}}}{N_{\mathrm{TPB}}}$$

where *N*_*SM*_ is the number of SMs, *N*_*MRT*_ is the maximum number of resident threads per SM supported by the GPU, and *N*_*TPB*_ is the number of threads per thread block configured by the user. The dynamic scheduling parallelization works as follows. All sequence profiles are organized into sequence profile blocks, each of which has as many sequence profiles as the number of threads in a thread block. Subsequently, *N*_*T*_ thread blocks are launched to perform the computation, where a thread block processes a sequence profile block at a time. When a thread block finishes its current computation, this thread block will dynamically obtain an unprocessed profile block. This operation is done by the atomic addition function *atomicAdd*() on global memory, which increments the index of global profile blocks. In our algorithm, both static scheduling and dynamic scheduling have used a thread block size of 64.

Our evaluation has found that dynamic scheduling performs slightly better than static scheduling when using the Swiss-Prot database (with large sequence length deviation), whereas the latter seems slightly better in the ideal case where all sequences are of equal lengths. Based on the above observations, we have decided to employ the static scheduling approach for databases with very small sequence length deviation (by default when the standard deviation does not exceed 1% of the mean) and the dynamic scheduling approach for all others.

#### Query profile variant

Given a query *S* defined over an alphabet Σ, a query profile is defined as a numerical string set *P* = {*P*_*r*_ | *r* є Σ}, where *P*_*r*_ is a numeric string comprised of substitution scores required for aligning the whole query to any residue in Σ. The space complexity of the query profile can be calculated as *O*(|*S*|×|Σ|). In our algorithm we have employed the sequential-layout query profile [16], which defines the *i*^th^ element of *P*_*r*_ as *M*(*r*, *S*[*i*]), 1≤*i*≤|*S*|. The query profile is stored in texture memory and has been packed in the same way as in CUDASW++ 2.0 to reduce the number of texture fetches.

**Figure 3:**
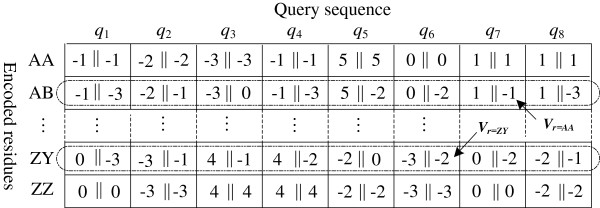
**An example query profile variant using *K*=2.** Operation *a*||*b* means concatenating 8-bit substitution scores *a* and *b* to form a short integer data.

To facilitate GPU SIMD parallelization, we have derived a variant of a query profile. By enumerating all residues in Σ, we define a query profile variant as a numerical set *V* = {*V*_*r*_ | 0≤ *r*<|Σ|^*K*^} of |Σ|^*K*^ entries, where *V*_*r*_ is a vector of *K* substitution scores and *r* is an integer corresponding to the permutation of any *K* residues in Σ. *V*_*r*_ stores all substitution score vectors for aligning the whole query to the *K* residues corresponding to *r*. The space complexity of a query profile variant can be calculated as *O*(|*S*|×|Σ|^*K*^). Like the query profile, this variant is also stored in texture memory. When *K* = 4, each element of *V*_*r*_ can be directly used in our quad-lane SIMD computation. However, the memory footprint is considerable even for short protein queries (this pressure can be significantly alleviated for DNA sequences due to their small alphabet size), and will cause more texture cache misses as the query length increases. In order to improve speed for long queries, our algorithm therefore uses *K*=2. We represent each element of *V*_*r*_ using the *short integer* data type, since the range of the *char* data type is generally large enough to store a substitution score. Figure 3 shows an example query profile variant using *K*=2. Similar to the query profile, the variant has also been packed by representing four consecutive elements of each *V*_*r*_ using the *short4* data type. In this way, the variant can reduce the number of texture fetches by half compared to the query profile. On the other hand, to compute each cell vector (see Figure 2), we have to extract and concatenate the substitution scores, from either query profile or the variant, to generate a substitution score vector. This requires some additional bitwise operations in our implementation. In this case, we can save six bitwise operations for each cell vector by using the variant, instead of the query profile. This makes great sense in terms of speed, considering that each cell vector requires only several assembly instructions as shown in Figure 2.

Our algorithm employs both the query profile and its variant. In general, for short queries, more performance gains can be realized from the query profile variant because it can reduce the number of texture fetches by half and use fewer bitwise operations per cell as mentioned above. However, for longer queries, a query profile becomes superior due to its much smaller memory footprint and less texture cache miss. Thus, we have calculated a query length threshold *Q* to decide whether to use the query profile or the variant. For the Kepler architecture, texture fetches are cached by the aforementioned read-only cache and L2 cache. Since the L2 cache is usually much larger than the read-only cache, we have estimated *Q* from the L2 cache size as

(4) $$Q=\frac{L2\_\mathrm{cache}\_\mathrm{size}}{2{\left|\sum \right|}^{K}}$$

The estimation is empirical and works well in practice through our evaluations. *Q* is used in the dynamic scheduling parallelization to cope with more general databases. For static scheduling which is only applied to databases with small sequence length deviations, we have found that a query profile variant usually leads to superior speed.

## Results and discussion

### Experimental design

We used the GCUPS metric to measure the performance of the following algorithms: CUDASW++ 3.0 (v3.0.14), CUDASW++ 2.0 (v2.0.10), SWIPE (v2.0.5) and BLAST+ (v2.2.27). We used 20 protein queries of lengths ranging from 144 to 5,478 to search against two protein databases: the Swiss-Prot database (release 2012_11) and a simulated database of equal-length sequences. The accession numbers of all queries are: P02232, P05013, P14942, P07327, P01008, P03435, P42357, P21177, Q38941, P27895, P07756, P04775, P19096, P28167, P0C6B8, P20930, P08519, Q7TMA5, P33450, and Q9UKN1, listed in the ascending order of sequence length. The Swiss-Prot database consists of 191,240,745 amino acids in 538,585 sequences and has the largest sequence length 35,213. The simulated database comprises 200,000 sequences with each sequence of length 3,000, containing 600,000,000 amino acids in total.

All tests were conducted on a personal computer with an Intel *i*7 2700K quad-core 3.5 GHz CPU and 16 GB memory, running the Linux operating system (Ubuntu 12.04). All GPU-based tests are carried out on the aforementioned GTX680 and GTX690 graphics cards. GTX680 has a single GPU that contains 8 SMs (1,536 SPs and a clock rate of 1.06 GHz) and 2 GB memory. GTX690 consists of two GPUs, each of which contains 8 SMs (1,536 SPs and a clock rate of 1.02 GHz) and 2 GB memory. We turned off the error correcting code on both graphics cards and conducted all single-GPU evaluations on GTX680 as well as all dual-GPU evaluations on GTX690. For all tests, the wall clock times were used to compute the GCUPS performance of all evaluated algorithms.

The CUDASW++ 3.0, CUDASW++ 2.0 and SWIPE algorithms used the default scoring schemes due to their runtime independence of scoring schemes. BLAST+ used the scoring matrices BLOSUM62 (BL62) and BLOSUM50 (BL50), with the default gap open and extension penalties. We used four CPU threads for the CUDASW++ 3.0, SWIPE and BLAST+ algorithms, and used other parameters “-b 0 -v 0” for SWIPE and “-num_alignment 0” for BLAST+, respectively. CUDA toolkit 4.2 was used to compile CUDASW++ 2.0 and CUDASW++ 3.0.

### Evaluation on the Swiss-Prot database

**Figure 4:**
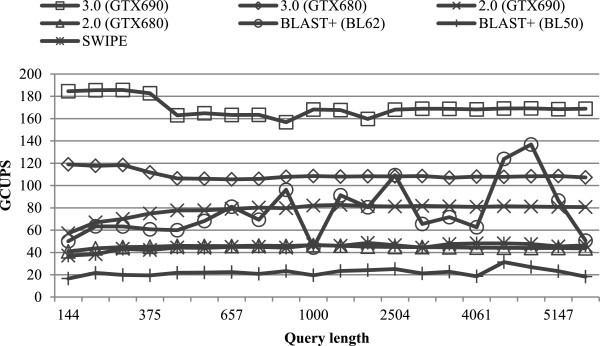
Performance comparisons on the Swiss-Prot database.

We first compared the performance of all evaluated algorithms by searching the 20 queries against the Swiss-Prot database. Figure 4 illustrates the performance of all evaluated algorithms for varying query lengths. For the Swiss-Prot database, CUDASW++ 3.0 employs the dynamic scheduling approach for all queries. On GTX680 (GTX690), CUDASW++ 3.0 yields an average performance of 109.4 (169.7) GCUPS, with a maximum of 119.0 (185.6) GCUPS. Highest performance is realized by short queries of lengths <400 due to the use of the query profile variant. In addition, a sudden performance drop can be observed as the curve moves to query length ≥400. This is because our CUDA kernel switches to the use of the query profile for longer queries, giving up the query profile variant.

Both CUDASW++ 2.0 and SWIPE achieve nearly constant performance for all queries. CUDASW++ 2.0 has an average performance of 44.8 (77.9) GCUPS on GTX680 (GTX690), while SWIPE yields an average performance of 45.0 GCUPS using 4 threads. CUDASW++ 3.0 is superior to both CUDASW++ 2.0 and SWIPE for every query, even if only using a single GPU. CUDADSW++ 3.0 on GTX 680 (GTX690) runs on average 2.4× (2.2×) faster than CUDASW++ 2.0 and 2.4 × (3.8×) faster than SWIPE, while gaining a maximum speedup of 2.9 (3.2) over CUDASW++ 2.0 and 3.2 (5.0) over SWIPE. BLAST+ shows performance fluctuations for different queries, especially in the case of BL62. Furthermore, BLAST+ is runtime sensitive to the scoring scheme used. It runs on average 3.4× faster using BL62 than BL50. On GTX690, CUDASW++ 3.0 is always superior to BLAST+ for each case, where the former achieves an average speedup of 2.4 and 7.8 (and a maximum of 3.8 and 11.1) over the latter using BL62 and BL50, respectively. On GTX680, CUDASW++ 3.0 outperforms BLAST+ using BL50 for all queries, gaining an average speedup of 5.1 and a maximum of 7.2. Compared to BLAST+ using BL62, CUDASW++ 3.0 gains an average speedup of 1.6 and a maximum of 2.4. However, our algorithm has a lower performance for two queries with the following accession numbers: P08519 and Q7TMA5.

### Evaluation on a simulated database

**Figure 5:**
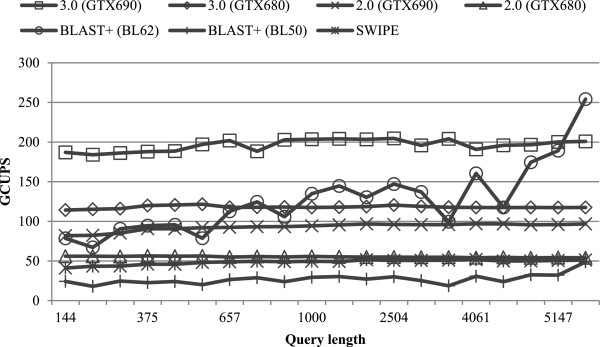
Performance comparisons on the simulated database.

In addition, we have employed the aforementioned simulated database to compare all algorithms. On this database, we can avoid the computation waste of CPU and GPU SIMD instructions as all alignments in all lanes will be completed at the same time, and can also avoid the computational imbalance between threads within a warp and a thread block. Figure 5 shows the performance of all evaluated algorithms on this simulated database.

Compared to the Swiss-Prot database, all evaluated algorithms are able to improve their average performance. On GTX680 (GTX690), CUDASW++ 3.0 achieves an average performance of 118.0 (196.2) GCUPS and CUDASW++ 2.0 of 55.2 (92.9) GCUPS. SWIPE improves its average performance to 48.6 GCUPS and BLAST+ to 126.9 and 27.1 GCUPS using BL62 and BL50 respectively. Similar to the Swiss-Prot database, CUDASW++ 2.0 and SWIPE produce nearly constant performance over all queries, while BLAST+ fluctuates. CUDASW++ 3.0 is still superior to CUDASW++ 2.0, SWIPE and BLAST+ using BL50 in each case. On GTX680 (GTX690), our algorithm gains an average speedup of 2.1 (2.1) over CUDASW++ 2.0, 2.4 (4.0) over SWIPE and 4.6 (7.6) over BLAST+ using BL50. Compared to BLAST+ using BL62, CUDASW++ 3.0 on GTX690 is superior for all queries except for the largest one, for which BLAST+ has a performance burst of up to 254.2 GCUPS. On average, CUDASW++ 3.0 on GTX680 can be considered on par with BLAST+ using BL62, but on GTX690 runs 1.7× faster.

### Other evaluations

In addition to the performance based on hybrid CPU-GPU parallelism, we have evaluated the performance of GPU-only CUDASW++ 3.0 by disabling CPU threads. By default, the GPU computation only supports subject sequences of lengths ≤3072 (as mentioned above) due to the limited GPU device memory. Longer subject sequences (>3072 residues) are distributed to the CPU. Hence, for this evaluation we created a new sub-database by extracting all sequences of lengths ≤3072 from the Swiss-Prot database. This new sub-database consists of 99.88% sequences and 98.41% amino acids of the original Swiss-Prot database. Using this sub-database, CUDASW++ 2.0 will only conduct the inter-task parallelization stage because all sequence lengths are ≤3072. In addition, we have disabled Stage (*iv*), which re-computes the very few alignments with indicative overflows on CPUs.

**Figure 6:**
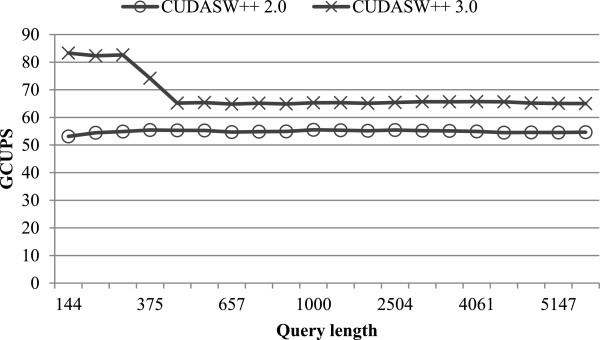
Performance comparison between GPU-only CUDASW++ 3.0 and 2.0.

Figure 6 shows the performance comparison between GPU-only CUDASW++ 3.0 and CUDASW++ 2.0 on the single-GPU GTX680. Due to the large sequence length deviation of the sub-database, the dynamic scheduling approach is automatically selected by CUDASW++ 3.0 for all queries. Similar to the case of using the original Swiss-Prot database, we have also observed a performance drop because of the switch from the query profile variant to the query profile. From the figure, GPU-only CUDASW++ 3.0 is superior to the CUDASW++ 2.0 for all queries, yielding an average speedup of 1.2 and a maximum speedup of 1.6. In addition, CUDASW++ 3.0 realized an average performance of 68.3 GCUPS and a maximum performance of 83.3 GCUPS, for all queries.

**Figure 7:**
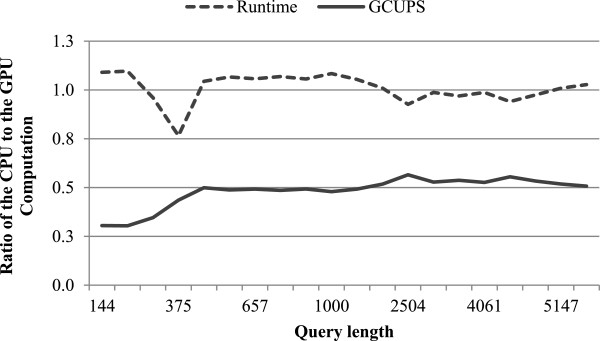
Relative performance of CPU SIMD computation to GPU SIMD computation.

Finally, we have evaluated the relative performance of CPU computation to GPU computation in Stage (*ii*), by searching all queries against the Swiss-Prot database on the GTX680. We have measured the runtimes of both the CPU and GPU computation and then calculate their performance from their respective workload and runtime. Figure 7 shows the performance ratio of the CPU to CPU computation in terms of runtime and GCUPS. From the figure, we can see that the runtime ratios of the CPU to GPU computation slightly fluctuate around 1.0 for all queries. This reflects that our workload distribution between the CPU and GPU computation are well balanced. In addition, for longer queries of lengths >400, the performance ratio of the CPU to GPU computation has also reached roughly stable values (about 1:2 on average).

## Conclusions

In this paper, we have presented CUDASW++ 3.0, a faster SW protein database search algorithm, which gains high speed by coupling CPU and GPU SIMD instructions and carrying out concurrent CPU and GPU computations. For the first time, we have investigated a GPU SIMD parallelization based on CUDA PTX SIMD video instructions. This parallelization enables us to gain more data parallelism beyond the SIMT execution model on CUDA-enabled GPUs. Performance evaluation reveals that our algorithm gains significant speedups over three other top-performing algorithms: CUDASW++ 2.0, SWIPE and BLAST+. On the popular Swiss-Prot database, our algorithm on GTX680 (GTX690) yields a speedup of up to 2.9 (3.2) over CUDASW++ 2.0, up to 3.2 (5.0) over SWIPE using 4 threads, and up to 7.2 (11.1) over BLAST+ with BL50 using 4 threads. With Hyper-Threading enabled, the performance of both SWIPE and BLAST+ against the Swiss-Prot database improves, albeit insignificantly. On average, compared to the aforementioned performance with 4 threads, the Hyper-Threading functionality can improve the performance by 12.8% and 34.0% for SWIPE and BLAST+ respectively, by using 8 threads. Despite designed for the SW protein database search, our algorithm has also presented a general computing framework for heterogeneous computing with CUDA-enabled GPUs and is expected to make contributions to other research problems.

## Abbreviations

BL50: BLOSUM50; BL62: BLOSUM62; CPU: Central Processing Unit; CUDA: Compute Unified Device Architecture; GPU: Graphics Processing Unit; GTX680: GeForce GTX 680; GTX690: GeForce GTX 690; FPGA: Field Programmable Gate Array; ISA: Instruction Set Architecture; PTX: Parallel Thread Execution; SSE: Streaming SIMD Extensions; SIMD: Single Instruction Multiple Data; SIMT: Single Instruction Multiple Thread.

## Competing interests

The authors declare that they have no competing interests.

## Authors’ contributions

YL contributed the idea of concurrent CPU and GPU execution, programmed the algorithm, performed the tests, analysed the results and drafted the manuscript; AW programmed the algorithm, analysed the results and revised the manuscript; BS contributed the idea of using PTX SIMD video instructions, participated in the algorithm optimization, analysed the results and revised the manuscript. All authors read and approved the final manuscript.
